# Pathogenic Triad in Bacterial Meningitis: Pathogen Invasion, NF-κB Activation, and Leukocyte Transmigration that Occur at the Blood-Brain Barrier

**DOI:** 10.3389/fmicb.2016.00148

**Published:** 2016-02-19

**Authors:** Shifu Wang, Liang Peng, Zhongtao Gai, Lehai Zhang, Ambrose Jong, Hong Cao, Sheng-He Huang

**Affiliations:** ^1^Department of Children's Medical Laboratory Diagnosis Center, Qilu Children's Hospital of Shandong UniversityJinan, China; ^2^Children's Hospital Los Angeles, Keck School of Medicine, University of Southern CaliforniaLos Angeles, CA, USA; ^3^Department of Clinical Laboratory, The Second Affiliated Hospital of Guangzhou Medical UniversityGuangzhou, China; ^4^Guangdong Provincial Key Laboratory of Tropical Disease Research, Department of Microbiology, School of Public Health and Tropical Medicine, Southern Medical UniversityGuangzhou, China

**Keywords:** pathogenic triad, bacterial meningitis, pathogen invasion, NF-κB activation, leukocyte transmigration, blood-brain barrier

## Abstract

Bacterial meningitis remains the leading cause of disabilities worldwide. This life-threatening disease has a high mortality rate despite the availability of antibiotics and improved critical care. The interactions between bacterial surface components and host defense systems that initiate bacterial meningitis have been studied in molecular and cellular detail over the past several decades. Bacterial meningitis commonly exhibits triad hallmark features (THFs): pathogen penetration, nuclear factor-kappaB (NF-κB) activation in coordination with type 1 interferon (IFN) signaling and leukocyte transmigration that occur at the blood-brain barrier (BBB), which consists mainly of brain microvascular endothelial cells (BMEC). This review outlines the progression of these early inter-correlated events contributing to the central nervous system (CNS) inflammation and injury during the pathogenesis of bacterial meningitis. A better understanding of these issues is not only imperative to elucidating the pathogenic mechanism of bacterial meningitis, but may also provide the in-depth insight into the development of novel therapeutic interventions against this disease.

## Introduction

Bacterial meningitis (BM) is one of the top ten causes of infection-related deaths worldwide (van de Beek et al., 2012). BM remains a major global challenge for people's health and wellbeing in twenty-first century. The mortality rates vary between 10 and 15% and are especially high in the developing countries. The incidence of BM is about five cases per 100,000 adults per year in the developed countries and may be 10 times higher in the developing countries (Brouwer et al., 2010). The predominant causative pathogens in adults are *Streptococcus pneumoniae, Neisseria meningitidis*, and *Listeria monocytogenes* which are responsible for about 80% of all cases (van de Beek et al., 2006; Brouwer et al., 2010), while group B Streptococcus (GBS), *S. pneumoniae, Escherichia coli, N. meningitidis*, and *Haemophilus influenza* type B cause about 90% of cases of BM in children globally (Galiza and Heath, 2009; Brouwer et al., 2010). The mortality rate of neonatal meningitis was considerably higher than those from other BM. Thus, bacterial meningitis is particularly devastating for newborns (Galiza and Heath, 2009); In addition, twenty to fifty percent of survivors can suffer permanent neurological sequelae, including seizures, deafness, hydrocephalus, cerebral palsy, and/or cognitive deficits, and so on Berardi et al. (2010). *S. pneumoniae* and *N. meningitidis* can cause the invasive infections at any age in both children and adults. Most cases of BM happen sporadically; only meningococcal infections may occur in epidemics (Lepage and Dan, 2013). So the prevalence of BM is still a huge threat to global public health security.

Patients with BM may experience the symptoms of vomiting, with the cardinal signs of meningitis alone, or in association with focal neurological deficit, or with encephalopathy. This variability is related to the physiopathological mechanisms depending on the nature of the infectious pathogens (Putz et al., 2013). Currently, the common mechanisms responsible for the modulation of the host response to meningitic infections are still not thoroughly understood, but overwhelming evidence from both clinical and preclinical research suggests that the most challenging issue of BM is the lack of understanding of the triad hallmark features (THFs) of this disease: pathogen invasion, NFκB activation and leukocyte transmigration that occur at the BBB (Huang et al., 2000; Kim, 2003; Radek et al., 2010; Takano et al., 2015). This review outlines the progression of these early correlated events contributing to BBB injury and CNS inflammation during the pathogenesis of BM.

## Methods

We sought to conduct a narrative review of the available literature focused on one or all of the three hallmarks of bacterial meningitis. To that end, a review of the English-language international medical literature was conducted using the terms [“bacteremia, BMEC, hallmarks, meningitis, NF-κB, blood-brain barrier (BBB), leukocyte transmigration”] to identify reported cases and studies of bacterial meningitis. Databases were searched from inception through October 2015.

### Pathogenic triad in bacterial meningitis: pathogen invasion, NF-κB activation, and leukocyte transmigration

**Figure 1:**
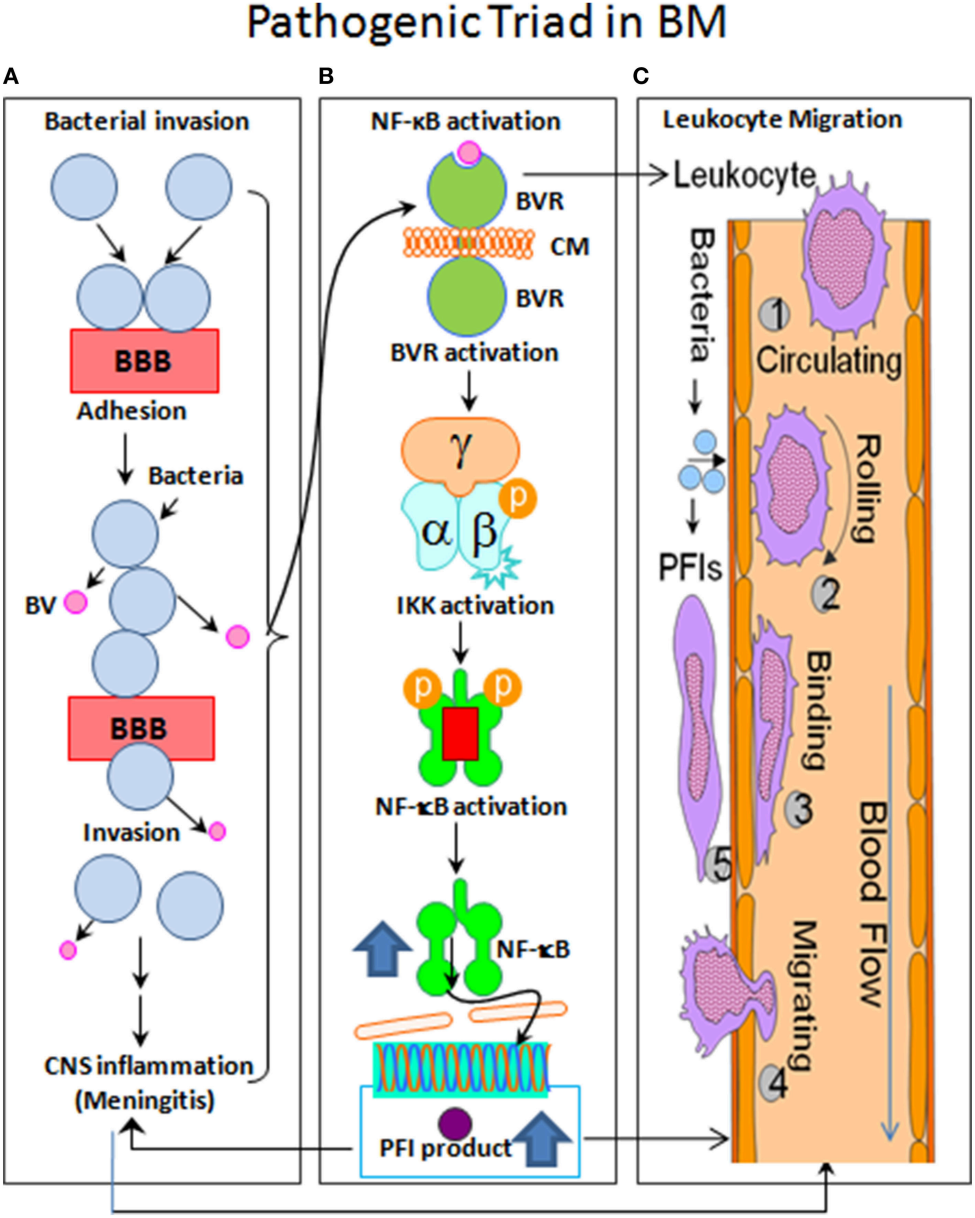
**Flow chart of the pathogenic triad in bacterial meningitis (BM). (A)** Bacterial pathogens adhere to brain microvascular endothelial cells (BMEC) that is the major component of the blood-brain barrier (BBB) and may then transcytotically pass to subendothelial tissues. **(B)** The nuclear factor-kappaB (NF-κB) is activated by bacterial virulence factors (BV) through their binding to BMEC membrane (CM) receptors (BVR), which can activate the enzyme IκB kinase (IKK) complex (α/β/γ). IKK, in turn, phosphorylates the NF-κB inhibitor IκBα, which results in dissociation of IκBα from NF-κB and eventual degradation of IκBα by the proteosome. The activated NF-κB is then translocated into the nucleus where it cooperatively activates gene transcription with other proteins such as coactivators and RNA polymerases. **(C)** The sequential steps are shown with numbers. (1), Leukocyte free in circulation and non-adhesive to endothelial cells; (2), Leukocyte tethered to endothelium and rolling under force of blood flow; (3), Leukocyte bound to endothelium and migrating via integrins and intercellular adhesion molecule-1 (ICAM-1); (4), Extravasation of leukocyte from blood vessel with the involvement of junctional adhesion molecule (JAM) and platelet endothelial cell adhesion molecule-1 (PECAM-1); and (5), Leukocyte migrates to source of infection or injury through integrins.

Over the past decades, the studies of bacterial meningitis caused by various pathogens revealed the importance and significance of bacterial virulence factors contributing to bacterial adhesion/invasion, NF-κB activation, and leukocyte transmigration through their interactions with host factors in brain microvascular endothelial cells (BMEC) (Huang et al., 2000; Chi et al., 2012; Figure 1). One of the most challenging issues is the lack of a comprehensive understanding of the molecular basis underlying all three of the interrelated hallmark features of this disease. The *in vitro* (BMEC) and *in vivo* (animal models) of the BBB, the two major methods, have been critical in dissecting the mechanisms underlying the pathogenic triad (Huang et al., 2000; Kim, 2003; Chi et al., 2012).

## Bacterial invasion across the blood-brain barrier

BBB is a specialized layer of BMECs that regulates the macromolecular traffic to maintain biochemical homeostasis in brain tissues. Due to the existence of specialized inter-endothelial junction complexes, the majority of microorganisms are unable to cross the BBB. To be successful as meningitic pathogens, they must possess traits that allow them to enter the blood stream (bacteremia) and to cross the BBB (meningitis). An exception is *S. pneumonia*, which can travel from the nasopharynx to cause meningitis without the intermediate step of the bacteremia and therefore avoids normal host defense mechanisms, crosses the BBB, and survives in cerebrospinal fluid (CSF) (Pizarro-Cerdá and Cossart, 2006; Figure 1). BM most frequently results from the bacteremia, such as GBS, *S. pneumoniae, E. coli* K1, *N. meningitidis, L. monocytogenes*, and *Mycobacterium tuberculosis* (Mtb) infection (Heckenberg et al., 2014), which are essential for the pathogen invasion across the BBB (Huang et al., 2000; Chi et al., 2012). Pathogen penetrations across the BBB is one of the most critical steps in the pathogenesis of BM, one of the hallmark features of this disease (Tracey, 2007; Peng et al., 2014).

### *Streptococcus spp*. invasion

The major pathogens of BM in children are *Streptococcus* spp., including GBS, *S. pneumonia* and *Streptococcus suis (S. suis)*. GBS is the most common bacterial pathogen that causes neonatal BM in the United States, Europe and Asia (Simonsen et al., 2014). In recent years, GBS has also emerged as a cause of serious infections including meningitis in the non-pregnant adult population (Skoff et al., 2009). In gaining access to the CNS, GBS displays an ability to cross the BBB. The penetration of BBB by bacterial pathogen reflects a complex interplay between host endothelium and microbial products. GBS interaction with glycosaminoglycan (GAG) promotes BBB attachment and bacterial entry into the CNS. The GAG binding property of the surface-anchored alpha C protein (ACP) is one contributor to this invasive phenotype (Chang et al., 2011). *S. suis* is also an emerging zoonotic pathogen that causes severe human infections with varied diseases/syndromes (such as meningitis), especially in countries such as Vietnam and Thailand (Feng et al., 2014).

*S. pneumoniae* is one of the most common causes of BM in adults, young adults and children older than a year (Castelblanco et al., 2014). It can also cause pneumonia and sepsis, often accompanied by strong inflammatory responses. *S. pneumoniae* expresses a sialidase (NanA) that contributes to mucosal colonization, platelet clearance, and BBB penetration. The *S. pneumoniae* NanA-stimulated supernatant of monocytes can increase human BMEC permeability. During sepsis and meningitis, the over-activation of inflammatory monocytes/macrophages plays an important role in the endothelial barrier dysfunction (Chang et al., 2012). The increased BBB permeability and meningeal TNF-α levels are also found in *S. pneumoniae*-challenged mice (Tsao et al., 2001). Yung-Chi Chang et al. also observed that supernatants collected from wild type *S. pneumoniae*-infected THP-1 cells can significantly increase the BMEC permeability of monolayer by a large indicator protein (horseradish peroxidase) compared to the supernatants collected from uninfected or NanA mutant *S. pneumoniae* infected THP-1 cells. These finding suggests that NanA is very important for *S. pneumoniae* invasion into the BBB through increasing monocytes' release of proinflammatory cytokines, with the potential downstream effects on endothelial cell permeability (Chang et al., 2012).

### *Escherichia coli* invasion

**Figure 2:**
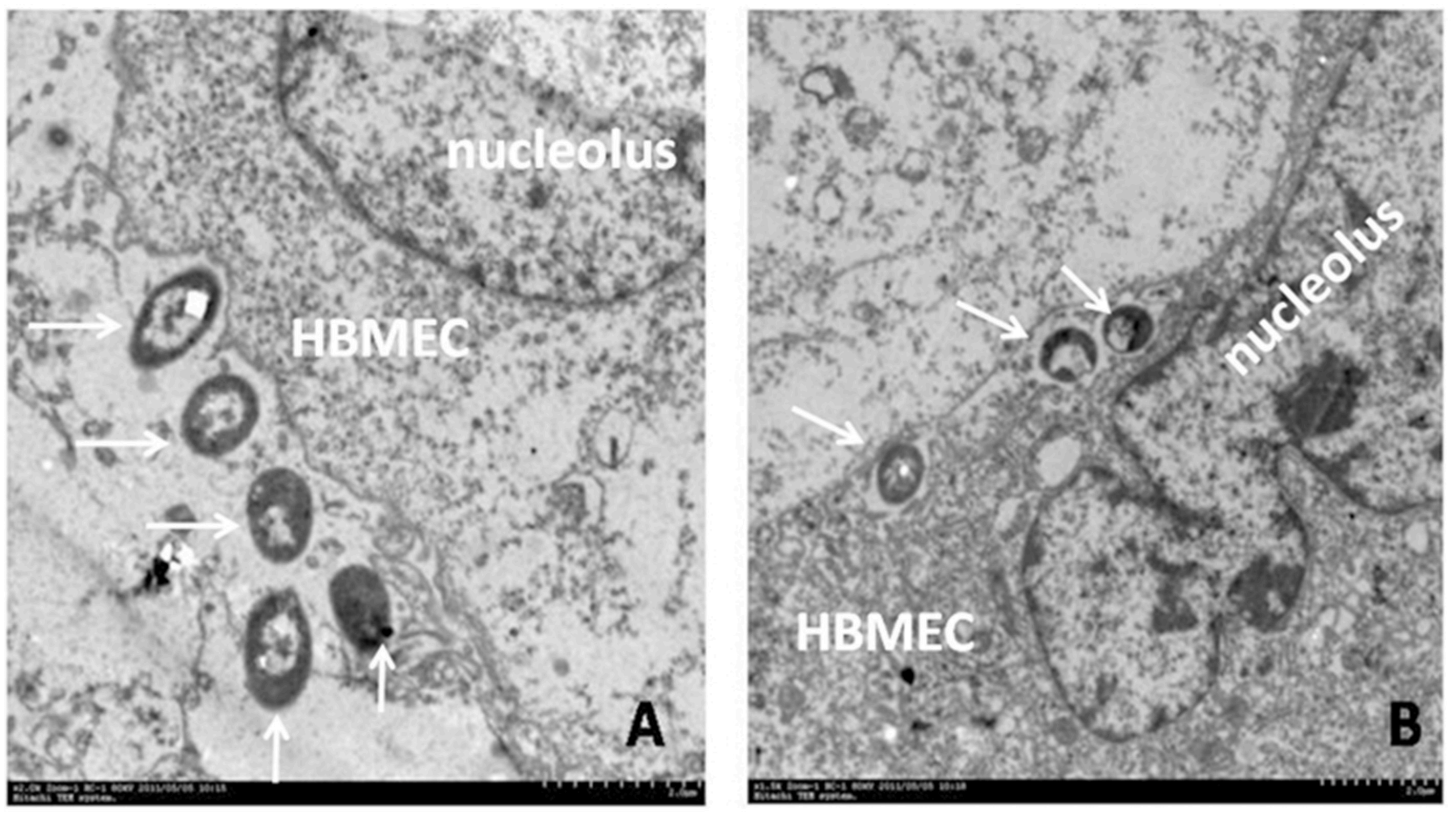
**Bacterial adhesion to and invasion of human BMEC (HBMEC)**. Transmission Electron Microscopy shows meningitic *E. coli* K1 (E44) adhesion to **(A)** and invasion across **(B)** human BMEC. *E. coli* closely contacted the human BMEC membrane and elicited its own uptake at the site of infection **(A)**. Intracellular bacteria are identified in membrane-bound vacuoles after 30 min of incubation **(B)**. Arrows indicate the processes of bacterial adhesion **(A)** and subsequent invasion **(B)**.

*E. coli* is the most common Gram-negative bacterial pathogen that causes neonatal meningitis. Several *E. coli* virulence factors (VFs), such as IbeA (invasion brain endothelial protein A), FimH (type 1 fimbrial tip adhesin) and OmpA (outer membrane protein A), and their receptors, vimentin, PTB-associated splicing factor (PSF), CD48 and gp96, have been identified and characterized (Chi et al., 2012). But only IbeA, IbeT, and FimH are genetically-unique virulence factors in meningitic *E. coli* which distinguish them from other *E. coli* determinants presenting in the normal flora. The *ibeA* gene is located on a genetic island GimA (Huang et al., 2001a), which is involved in the pathogenesis of microbial infections caused by neonatal meningitic *E. coli* (NMEC) and avian pathogenic *E. coli* (APEC) (Wang et al., 2011). IbeA could regulate expression of other virulence factors, such as FimH and auto-transporter adhesion, which mediated the adhesion of NMEC to the host cells (Cortes et al., 2008; Figure 2A). Furthermore, IbeA appears to increase the ability of *E. coli* K1 to invade BMEC via ligand receptor interactions (Huang et al., 2001b; Heckenberg et al., 2014; Figure 2B). It also has been demonstrated that nicotine is able to modulate the BBB permeability through the cholinergic α7 nAChR pathway. The α7 nAChR deficiency is protective against meningitic infections by the down-regulation of pathogen invasion. Our previous study has already confirmed that meningitic *E. coli* K1 penetration across the BBB is modulated by this receptor (Abbruscato et al., 2002; Chi et al., 2011).

### *Neisseria meningitidis* invasion

*N. meningitidis*, often referred to as *meningococcus*, is a major cause of meningitis and other forms of meningococcal disease such as meningococcemia, which is a life-threatening sepsis (Coureuil et al., 2012, 2014). It is also a frequent asymptomatic colonizer in human nasopharynx and only a very small fraction of infections proceed to a sustained bacteremia. Once it invaded in the bloodstream, *N. meningitidis* can either be responsible for a deadly septic shock leading to crossing the BBB or invading the meninges (Coureuil et al., 2012). The BBB is comprised of BMEC with tight junctions (TJs) and adherents junctions that are composed of type IV collagen, proteoglycan, laminin, glycoproteins, and astroglial end feet (Coureuil et al., 2014). Two host receptors, CD147 and the β2-adrenoceptor, are required for bacterial adhesion to and crossing of the BBB (Coureuil et al., 2014). The invasion of meningeal virtually is the consequence of an interaction between *N. meningitidis* and BMEC. This interaction, mediated by the type IV pili, is responsible for the formation of micro-colonies on the apical surface of BMEC. This interaction is followed by the activation of signaling pathways in BMEC, which leads to the formation of endothelial docking structures resembling those cells elicited by the interaction of leukocytes with BMEC during extravasations. The consequence of these signaling events induced by bacteria is the recruitment of intercellular junction components in the docking structure and the subsequent opening of the intercellular junctions (Coureuil et al., 2012). The depletion of TJs can lead to a paracellular route through the BBB and into the brain.

Virulence genes in *N. meningitidis* show that the carriage and invasive strains genetically belong to distinct populations. In the recent years, it has become more clear that the metabolic adaptation enables *N. meningitidis* to exploit the host resources, supporting the concept of virulence genes as a crucial capability in invasion (Coureuil et al., 2012). The *N. meningitidis* adhesin NadA was identified in about 50% of *N. meningitidis* isolates, which is closely related to the *Yersinia* adhesin YadA, the prototype of the oligomeric coiled-coil adhesin family (Nägele et al., 2011). NadA is known to be involved in cell adhesion, invasion, and induction of proinflammatory cytokines. Because of the enormous diversity of *N. meningitidis* cell adhesins, the analysis of the specific contribution of NadA in meningococcal host interactions is limited (Nägele et al., 2011; Coureuil et al., 2012).

### *Listeria monocytogenes* invasion

The Gram-positive bacterium *L. monocytogenes* can enter the human CNS and cause life-threatening meningitis. It is the third most common cause of community-acquired BM which occurs most frequently in elderly and immunocompromised patients (Koopmans et al., 2013). The disease is known to target immunocompromised individuals and is characterized by febrile gastroenteritis, infection of the fetuses in the pregnant women and CNS infections, such as meningitis and meningoencephalitis (Drevets and Bronze, 2008). During this process the pathogen has to invade and cross diverse cellular barriers involving the functions of the surface proteins Internalins A (InlA) and B (InlB). InlA and InlB are interdependently required for polar basolateral invasion by *L. monocytogenes* in a human model of the blood-cerebrospinal fluid barrier (Gründler et al., 2013).

### Other bacteria invasion

Other bacteria also can invade the BBB, such as *H. influenza, Klebsiella*, and *Pseudomonas aeruginosa*. Right now, the inclusion of the *H. influenzae* vaccine in the immunization programs of many countries has greatly reduced this invasive disease. Mtb, the causative agent of tuberculosis (TB), is one of the world's leading infectious causes of death. Tuberculosis infects about 1.3 million new patients and causes 450,000 deaths among children annually (Zucchi et al., 2013). Tuberculosis meningitis (TBM) is a serious global health problem with a delayed initiation awareness, which is often attributable to the use of slow or relatively insensitive conventional diagnostic tests. Besides, TB has been shown to be associated with high mortality rates in patients with TBM (Kim et al., 2010; Zucchi et al., 2013). It is clear that Mtb invades the CNS, but the underlying mechanisms of the interplay between the host and microbial organisms are poorly understood. Theoretically, Mtb may cross the BBB as free (extracellular) organisms or via the infected monocytes/neutrophils. Mtb was found to invade and traverse the BMEC monolayers, which suggests that Mtb triggers its own uptake from human BMECs. In addition, pathogenic Mtb strains H37Rv and CDC 1551 can invade and traverse human BMEC monolayers more than the nonpathogenic species (Kim et al., 2010).

In conclusion, the specific bacterial virulence factors for meningeal pathogens include specialized surface components that are crucial for adherence to the epithelium, the evasion of local host defense mechanisms, and subsequent invasion of the bloodstream for the pathogenesis of BM, which is the penetration of the extracellular pathogens across the BBB. It is very clear that it is not only the encoded repertoire of adhesins and invasions that allows the bacteria to adhere to the host cells and to evade the innate and acquired immunity of the host, respectively, but foremost the bacteria can exploit the host resources to their advantage through metabolic adaptation, which also plays a central role in pathogen crossing the BBB (Abu Kwaik and Bumann, 2013).

## NF-κB signaling and its crosstalk with type 1 interferon pathways in bacterial meningitis

**Figure 3:**
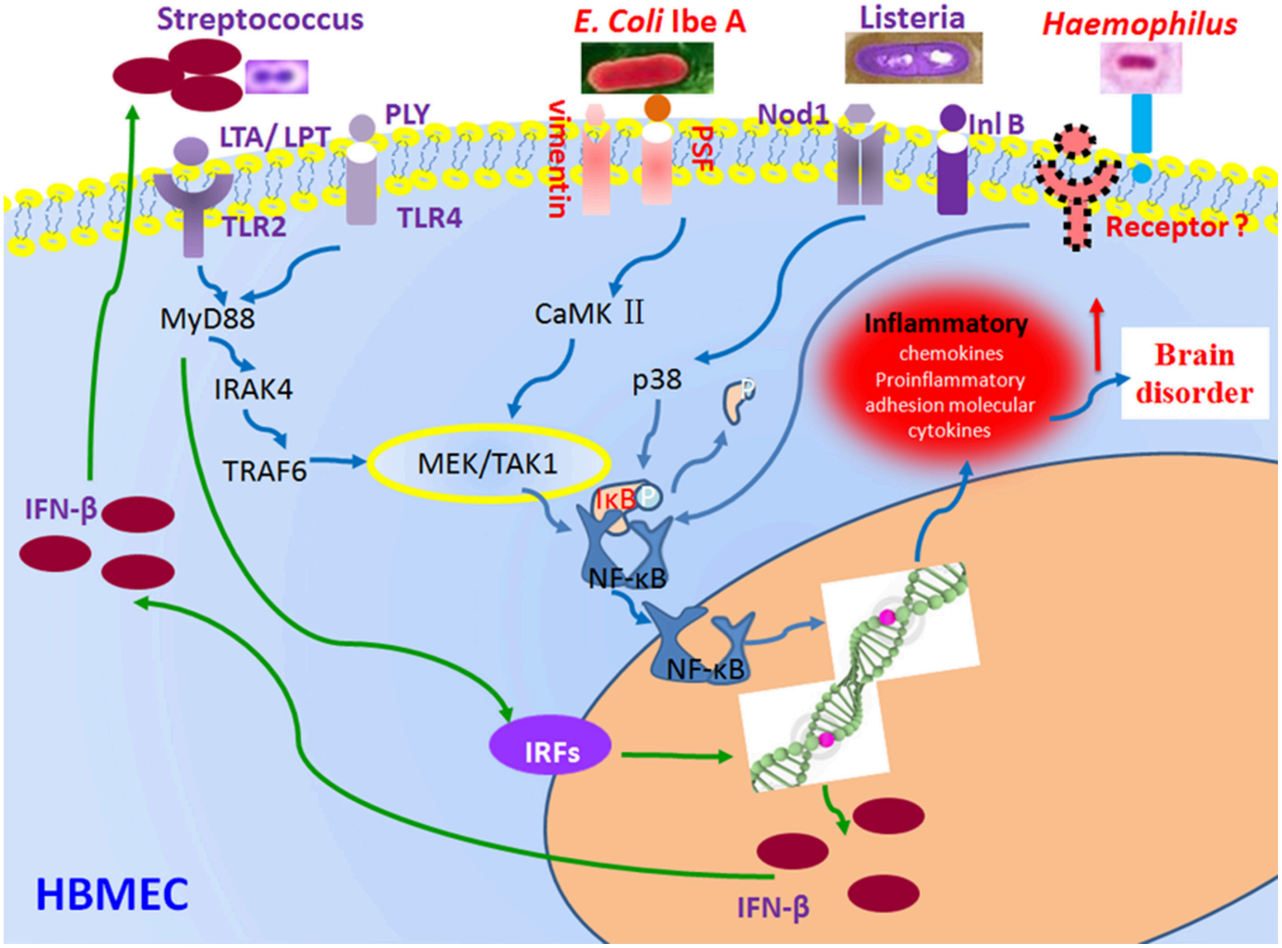
**Regulation of NF-κB and type 1 IFN signaling pathways by bacterial effectors**. Bacterial infection triggers receptor-dependent activation of the signaling pathways at the host cell membrane. Receptor activation triggers signaling protein phosphorylation, which in turn activates the nuclear factor-kappaB (NF-κB) pathways via activation of the IκB kinase (IKK) complex and up-regulate interferon-regulatory factor (IRF) family of transcription factors through MyD88-mediated signaling. *E. coli* K1 IbeA-binding proteins Vimentin and PTB-associated splicing factor (PSF) act in concert to activate NF-κB. NF-κB activation induced by *S. pneumoniae* depends on the host factors toll-like receptors 2 (TLR2) and 4 (TLR4), and their ligands lipoteichoic acid (LTA) and lipopolysaccharide transport (LPT) (pneumococcal cell wall compounds). The NF-κB activation induced by *L. monocytogenes* depends on the Nucleotide-binding oligomerization domain-containing protein 1 (Nod1) and internalin B (InlB) receptor. The two coordinated signaling pathways result in production of inflammatory factors (NF-κB) and IFN-β (type 1 IFN signaling).

Once the bacteria invade the local tissue barrier and the BBB, most pathogens can activate the transcription factor NF-κB that is another hallmark feature of BM, resulting in high levels of cytokines and inflammatory cytokines in the blood and cerebrospinal fluid (CSF), a result of the stimulation of phagocytic cells. NF-κB proteins consist of five different members, p65/RelA, c-Rel, RelB, NF-κB2/p52, and NF-κB1/p50, sharing a Rel homology domain that mediates DNA binding and dimerization (Chi et al., 2012). In resting cells, NF-κB is trapped in the cytoplasm by inhibitory IκB proteins. The NF-κB activation process is induced by phosphorylation of serine residues on the IκB proteins, which are then subjected to ubiquitination and proteasomal degradation. NF-κB has often been called a central mediator of the human immune response because many microbial pathogens, including meningitic bacteria, can activate this transcription factor that regulates the expression of inflammatory cytokines, chemokines, immune-receptors, and cell adhesion molecules (Pahl, 1999). The generation of intense inflammation in the subarachnoid space in response to the bacterial meningitis contributes to brain dysfunction and neuronal injury in BM. Microglia, the major immune effectors cells in the CNS, was activated by bacterial components to produce proinflammatory immune mediators. Furthermore, the level of NF-κB was higher in CSF of patients with BM than in those of patients with aseptic meningitis (Ichiyama et al., 2002). The NF-κB activation in CSF cells of patients with meningitis tended to be correlated with the CSF interleukin-6 (IL-6) concentration. CSF cells produced more proinflammatory cytokines in BM than in aseptic meningitis through NF-κB activation. The increased NF-κB activation in CSF cells could indicate severe inflammation in the CSF (Ichiyama et al., 2002; Figure 3). It has been shown that the production of type I interferons (IFNs) could be induced by *S. pneumoniae* through activation of the type 1 IFN signaling pathways in the host cells and that type I IFNs could regulate resistance and chemokine responses to bacterial infection in an autocrine/paracrine manner (Fang et al., 2014). The transcription of type 1 IFNs is modulated by a number of regulatory factors, including MyD88 (Wu et al., 2015). A recent study shows that the crosstalk between the type I IFNs and NF-kB pathways may an important role in virus-induced susceptibility to bacterial superinfection (Schliehe et al., 2015). These findings suggest that the two related signaling pathways may coordinately contribute to the pathogenesis of BM.

### NF-κB activation in bacterial meningitis caused by different bacteria

*E. coli* is the most common gram-negative bacterium causing neonatal sepsis and meningitis, 20–50% of survivors can suffer permanent neurological sequelae including deafness, seizures, hydrocephalus, cerebral palsy, and/or cognitive deficits (Galiza and Heath, 2009; Berardi et al., 2010). Recent studies have shown that IbeA-induced NF-κB signaling induced by meningitic *E. coli* K1, which can be blocked by an inhibitor of NF-κB (caffeic acid phenethyl ester CAPE), requires two IbeA-binding proteins, the primary receptor vimentin and the co-receptor PSF (Chi et al., 2012). Vim may form a complex with IkB, NF-κB and tubulins in the resting cells. A dissociation of this complex could be increased by IbeA in a time-dependent manner while the activation of ERK is entirely dependent on PSF. Moreover, IKK **α/**β phosphorylation is completely abolished in human BMECs lacking vimentin and PSF. Alpha7 nAChR-mediated calcium signaling is required for the interactions between IbeA and its receptors (vimentin and PSF), suggesting the involvement of α7 nAChR in the NF-κB signal transduction pathway (Chi et al., 2011; Heckenberg et al., 2014). All of them have demonstrated that the IbeA/vimentin mediated signaling is essential for NF-κB activation and PMN transmigration across the BBB, two of the three hallmark features of BM (Ichiyama et al., 2002; Che et al., 2011; Schliehe et al., 2015; Figure 3). FimH adhesion also activated the murine microglial cell line, BV-2, which resulted in the production of nitric oxide and the release of TNF-a. Mitogen-activated protein kinases, ERK and p-38, and NF-κB were involved in FimH adhesin-mediated microglial activation. These findings suggest that FimH adhesion also contributes to the CNS inflammatory response by virtue of activating microglia in *E. coli* meningitis through the NF-κB pathway (Lee et al., 2005).

*S. pneumoniae*, the major cause of community-acquired pneumonia and BM, has been shown to transiently invade epithelial and endothelial cells. After *S. pneumoniae* reaches the subarachnoid space, it multiplies rapidly and releases compounds, such as cell wall fragments, lipoteichoic acid (LTA), lipoproteins (LPT), pneumolysin (PLY), and peptidoglycan (Mook-Kanamori et al., 2011). TLR2 is activated by pneumococcal cell wall compounds, LTA and LPT. TLR4 is activated by PLY (Hanke and Kielian, 2011; Koppe et al., 2012). Both of TLR2 and TLR4 transmit their signals through a common intracellular adapter protein known as myeloid differentiation factor 88 (MyD88) (Koppe et al., 2012; Wu et al., 2015). The deficiency of this intracellular adapter protein in children can lead to increase their susceptibility to invasive meningitis (von Bernuth et al., 2008). MyD88 plays an important role in regulation of type 1 IFNs (Wu et al., 2015) and the IL-1 receptor-associated kinase-4 (IRAK4) (Kawai and Akira, 2007). After IRAK4 has been phosphorylated, it is dissociated from MyD88 and interacts with tumor necrosis factor receptor associated factor 6 (TRAF6) (Adhikari et al., 2007). TRAF6 stimulates the transforming growth factor β-activated kinase (TAK1). Thus, TAK1 activates IKK (Inhibitor of IkB kinase), which lead to the destruction of IkB and the subsequent activation and nuclear translocation of NF-κB (Malley et al., 2003; Kenneth et al., 2013). NF-κB is also an important transcriptional activator of various inflammatory genes implicated in neuronal pathogenesis and in the production of cytokines and chemokines (Che et al., 2011; Barichello et al., 2013; Figure 3).

*L. monocytogenes* is a facultative intracellular microorganism, which has the ability to invade most host cells including epithelial and endothelial cells (Regan et al., 2014). NF-κB is strongly activated in endothelial cells of mice infected by haemolytic *L. monocytogenes*, as opposed to non-haemolytic isogenic mutants. Kayal et al. have found that listeriolysin O (LLO)-deficient mutants did not induce the activation of human umbilical vein endothelial cells (HUVEC), as opposed to the mutants inactivated in the other virulence genes. Adhesion molecule (ICAM-1 and E-selectin) expression and NF-κB activation were fully restored by the strain of *Listeria innocua* transformed with the *hly* gene encoding LLO. LLO is involved in NF-κB activation in transgenic mice carrying an NF-kB-responsive lacZ reporter gene that were injected the purified LLO intravenously, thus it can induce the stimulation of NF-κB in endothelial cells of blood capillaries. It has been confirmed that LLO secreted by *L. monocytogenes* has the ability to serve as a potent inflammatory stimulus to induce NF-κB activation in HUVEC (Kayal et al., 1999). Recently, two members of a novel class of pattern recognition receptors, the cytosolic proteins nucleotide-binding oligomerization domain 1 (Nod1)/CARD4 and Nod2/CARD15, have been found to interact with the cell wall peptidoglycan. Nod1 is composed of peptidoglycan containing meso-diaminopimelic acid (Chamaillard et al., 2003). Nod1-overexpression experiments demonstrated that Nod1 is critically involved in chemokine secretion and NF-κB activation initiated by *L. monocytogenes* in HUVEC. Opitz et al. have demonstrated that Nod1 mediates activation of p38 MAPK and NF-κB, which contributes to IL-8 production in HUVEC infected with invasive *Listeria*, but TLR2 is not crucial for *Listeria*-induced IL-8 production in HUVEC (Opitz et al., 2006).

NF-κB activation also plays important roles in other BM, such as *H. influenza* encephalitis. It can induce the inflammatory responses through the activation of NF-κB via two distinct signaling systems, NIK-IKKα/β-IκBα and MKK3/6-p38 pathways. Glucocorticoids synergistically enhance *H. influenza*-induced TLR2 up-regulation likely via a negative cross-talk with the inhibitory p38 MAPK (Shuto, 2013; Figure 3).

### The role of other compounds and physical factors effect on NF-κB activation in bacterial meningitis

N-acetylcysteine (NAC), neuroprotective in animal models of acute brain injury caused by BM, has been shown to inhibit TNF-α-induced endothelin-1 (ET-1) upregulation independent of an effect on NF-κB pathway activation. Several lines of evidence have shown that NAC has the ability to inhibit the ET-1 upregulation through the inhibition of mitogen- and stress-activated protein kinase, a kinase involved in modulating the nucleosomal response in early gene induction by mitogenic and stress induced signals (Sury et al., 2006). NAC can also inhibit the TNF-α-induced rise in MSK1 and MSK2 kinase activity through the activation of NF-κB, while siRNA knock-down experiments showed that MSK2 is the predominant isoform involved in TNF-α-induced ET-1 upregulation (Sury et al., 2006).

Lipoteichoic acid (LTA), a component of Gram-positive bacteria cell wall, has been found to be elevated in the CSF of patients suffering from meningitis, it can induce proMMP-9 expression via the sequential activation of TLR2/MyD88, c-Src, PDGFR, PI3K/Akt, ERK1/2, IKKa/b, and NF-κB, leading to the promotion of RBA-1 cell migration. It suggests that LTA-induced proMMP-9 expression and cell migration through the NF-κB in astrocytes might play a crucial role in the progression of the CNS inflammatory diseases upon infection with Gram-positive bacteria (Hsieh et al., 2010).

In conclusion, NF-κB activation is one of the hallmark features of BM (Chi et al., 2012). As NF-κB is a central mediator of the human immune response (Constantoulakis et al., 2010), it is well-known that NF-κB signaling relies on the targeting of IkB (inhibitor of NF-κB) subunit to the proteasome to allow NF-κB to translocate from the cytosol to the nucleus, where it activates transcription of proinflammatory cytokine genes, which are essential to mount a protective immune response and host defense. The innate immune response in vertebrates is the first defense line against invading microorganisms (Constantoulakis et al., 2010). The cytoplasm activation and nuclear translocation of NF-κB represent a new paradigm in pathogen-induced signal transduction and lead to the development of novel strategies for the prevention and treatment of BM.

## Leukocyte transmigration into the central nervous system

Leukocytes are cells of the immune system which defend the body against both infectious diseases and foreign materials. The transmigration of leukocytes across the BBB into the CNS is another hallmark feature of BM (Zwijnenburg et al., 2006). Bacterium causes secretion of proinflammatory cytokines followed by the recruitment of leukocytes into the CNS. It is a key aspect of the protective response of leukocytes against invading pathogens. But in recent years, evidence suggests that leukocytes also contribute importantly to inflammation of the brain in BM (Zwijnenburg et al., 2006). The transmigration of leukocytes into the CNS through BBB can also lead to the disastrous consequences for people with BM (Zwijnenburg et al., 2006; Weber and Tuomanen, 2007).

**Figure 4:**
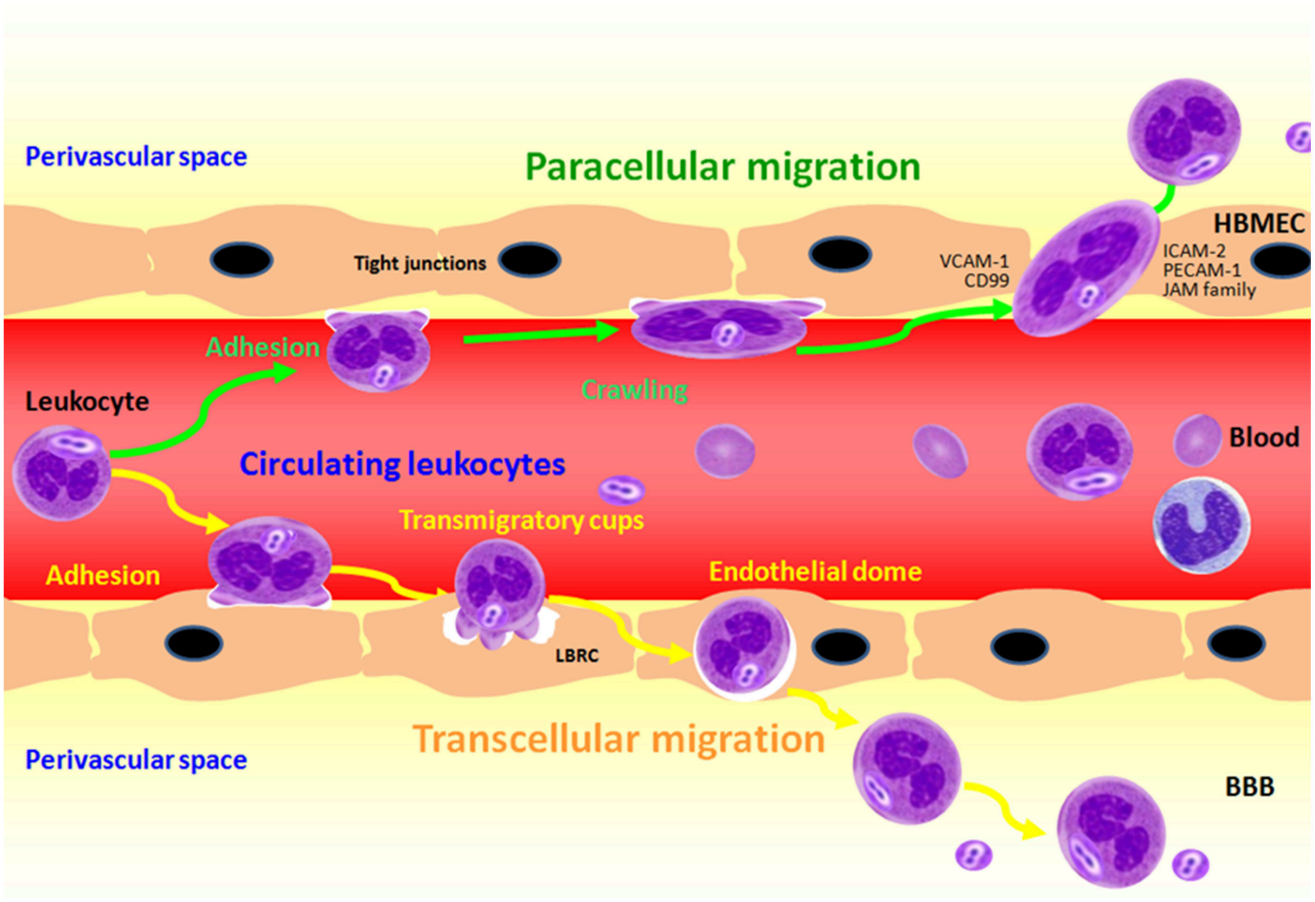
**Transendothelial migration of polymorphonuclear neutrophil (PMN) across BMEC in response to meningitic infections caused by bacterial pathogens**. PMN adhesion and crawling from the vascular lumen are the firstly two steps to close to the endothelial cells, and then PMN transmigration across the BBB through both the paracellular and transcellular pathways.

Once the bacteria invaded the tissue barriers, the leukocytes were quickly mobilized and transmigrated across the vascular endothelium. Strengthening and spreading adhesion, crawling intravascular, the leukocytes emigrate from the vascular lumen by passing between (paracellular) or through (transcellular) the endothelial cells (Figure 4). Both the two forms of transmigration may occur rapidly (within minutes) (Ley et al., 2007; Schmidt et al., 2011). *In vitro*, data has demonstrated that transcellular migration takes place in under a minute (Cinamon et al., 2004). It is still unclear, however, as to what mechanisms determine whether the neutrophils transmigrate via the paracellular or transcellular pathway (Wittchen, 2009; Muller, 2011) and the transcellular migration route, where the leukocytes overcome the TJs migrating between the cells involving a zipper-like mechanism (Chin and Parkos, 2007), and where the leukocyte migrates through the barrier-forming cell itself, respectively (Engelhardt and Wolburg, 2004). It has been demonstrated that leukocytes are able to transmigrate across the endothelium by using both paracellular and transcellular pathways. PMNs and monocytes may migrate in different way in a human blood-cerebrospinal fluid barrier model after BM (Wagner and Frenette, 2008; Steinmann et al., 2013).

### Transcellular migration of leukocytes through the blood-brain barrier

The migration of leukocyte across BBB is a “double-edge sword.” The recruitment of leukocyte into the CNS by transmigration across the BBB is not only crucial for host defense against BM, but it also responsible for the significant CNS tissue damage, which results in devastating neurologic sequelae (van der Flier et al., 2003). Although the adhesive interactions between transmigrating leukocytes and endothelial cells have been well known, the underlying mechanisms for diapedesis of leukocytes (extravasation) are largely unknown. Recent studies have showed that blood lymphocytes and neutrophils preferentially can transmigrate across brain endothelial cells via a transcellular route (Carman, 2009). This notion is supported by the recent finding of Che et al. that transcellular migration of PMN across BMEC is induced by meningitic *E. coli* K1 (Che et al., 2011). Previously it has been demonstrated that PMN preferentially transmigrate via the transcellular route through the primary porcine choroid plexus epithelial cells. PMN was raised to the infection of the non-BBB site that has been widely studied and well-known. However, because of the specificity of the BBB and leukocyte components, the adhesion and transmigration rates of PMN are faster those of lymphocytes. It has also been shown that PMNs transmigrate across the BBB in BM caused by GBS, *S. pneumoniae, E. coli, N. meningitidis* through the transcellular pathway (Doran et al., 2003; Doulet et al., 2006; Che et al., 2011). Our recent studies have demonstrated that the IbeA/Vim mediated signaling is essential for NF-κB activation and PMN transmigration across the BBB, two of three hallmark features of BM (Che et al., 2011; Chi et al., 2011, 2012).

Leukocyte adhesion induces the formation of pro-adhesive sites at the plasma membrane termed endothelial adhesive platforms, which are specialized tetraspanin-enriched microdomains that express high levels of intercellular adhesion molecule (ICAM-1) and vascular cell adhesion molecule 1(VCAM-1)(Barreiro et al., 2008; O'Carroll et al., 2015). In addition, BMEC form “docking structures” or “trans-migratory cups,” which are projections rich in ICAM-1, VCAM-1, cytoplasmic ERM (ezrin, radixin, and moesin) proteins and cytoskeletal components (such as vinculin, α-actin and talin-1) (Barreiro et al., 2002; Carman and Springer, 2004). These “docking structures” can facilitate the transmigration process through either the transcellular or paracellular pathway by surrounding the neutrophils, and then generate redistribution of the surface integrin leading to a conformational change that assists with the migratory process (Dimasi et al., 2013; Figure 4). Che et al. have recently defined *E. coli* K1-induced adhesive interactions between transmigrating leukocytes and brain endothelial cells in a manner dependent on the IbeA receptor vimentin. ICAM-1 and CD44 play a role in the leukocyte transmigration process during *E. coli* meningitis (Che et al., 2011). Mamdouh et al. have shown that there exists an intracellular endothelial structure, known as the lateral border recycling compartment (LBRC), which is critical to the transmigration process of monocytes (Mamdouh et al., 2003, 2008). The LBRC is a reticulum of interconnected tubule vesicular structures that lies just beneath the plasma membrane adjacent to the endothelial cell borders. Diapedesis of leukocytes (monocytes and neutrophils) across endothelial cells is required for many, but not all, inflammatory responses. This process depends on the homophilic interaction between platelet endothelial cell adhesion molecule 1 (PECAM-1) on the leukocytes (monocytes and neutrophils) and PECAM at the endothelial cell border (Mamdouh et al., 2008). LBRC is also required for transmigration of lymphocytes but it is independent of PECAM-1, suggesting that trafficking of membrane from the LBRC to surround leukocytes is a common mechanism for leukocyte transmigration across the endothelial cells.

As previously discussed, the factors that induce paracellular or transcellular migration of neutrophils across the BBB have not been defined. It has been speculated that neutrophils will migrate via the transcellular pathway in vascular areas where endothelial junctions (EJ) are tight, such as the blood-brain barrier, although this is inconsistent with the rates of transcellular migration that occur in regions with leaky junctions (Carman and Springer, 2008). As with paracellular migration, where the neutrophil is directed toward the inter-endothelial junctions, transcellular migration also appears to involve into the selection of appropriate migration sites by the neutrophils. *In vitro* studies have demonstrated that migrating leukocytes probe the endothelial surface in search of permissive sites for transcellular migration, using structures known as invadosome-like protrusions (ILPs) (Cinamon et al., 2004). The ILPs can invaginate into the surface of the endothelium in search of an area with minimal resistance, as this will provide the most efficient route for passage through the cell. Upon locating such a site, the ILPs progressively extend until they ultimately breach the basal endothelial membrane. Intracellular membrane vesicles located within the endothelial cytoplasm have been observed both *in vivo* and *in vitro* to become enriched around the site of the invagination of ILPs and are thought to assist in leukocyte migration by creating a “gateway” through the body of the endothelial cell (Dvorak and Feng, 2001; Carman et al., 2007). Mamdouh et al. also recently reported that the LBRC is critical for the transcellular migration of neutrophils (Figure 4 transcellular), similar to its involvement in paracellular migration with the recycling of CD99, PECAM-1 and the junctional adhesion molecules (JAMs) involved in this process (Mamdouh et al., 2009).

### Paracellular migration of leukocyte across the blood-brain barrier

Whereas, the mechanisms responsible for paracellular or transcellular migration are not well understood, one important determinant is the display of specific endothelial markers that indicate the most efficient method of transmigration under the prevailing conditions (Dimasi et al., 2013; Figure 4 paracellular). For example, there is evidence that ICAM-2 on the endothelial cell surface directs neutrophils toward the endothelial junctions (EJ) through binding to LFA-1, and then promote the paracellular migration (Woodfin et al., 2009). Another important feature of the early stages of the paracellular migration pathway is the reduced strength of the EJ, which is primarily mediated through redistribution of the molecule vascular endothelial adhesion (Shaw et al., 2001; Alcaide et al., 2009). In addition, the integrity of the EJ is compromised by the increased levels of intracellular endothelial Ca^2+^, the consequence of which is to increase the endothelial cell contraction via the activation of the myosin light-chain kinase (Huang et al., 1993). Upon localizing to the site of paracellular migration, a range of molecules expressed within the VE play their roles through the endothelial cell. These molecules include the JAMs family members JAM-A, JAM-B and JAM-C, PECAM-1, endothelial cell-selective adhesion molecule (ESAM) and CD99 (Ley et al., 2007; Woodfin et al., 2010; Schmidt et al., 2011), and so on.

Mononuclear cells in circulating blood supply peripheral tissues with macrophage and dendritic cell precursors that also directly contribute to immune defense against microbial pathogens (Ichiyama et al., 2009). Type 1 IFN signaling is required for macrophage-mediated inflammatory response through up-regulation of IL-18 induced by *S. pneumonia* (Fang et al., 2014). IFN-β, one of the type 1 IFNs, plays a role in Group A Streptococcus (GAS)-induced inflammatory response in macrophages. In addition to IFN-β signaling, MyD88-JAK1-STAT1 complex formation and increased SOCS-1 expression in GAS-infected macrophages may be more conducive to rapid bacterial infection and evasion of host immunity. Early expression of SOCS-1 is affected by NF-κB activation through reduction of STAT1 expression levels (Wu et al., 2015). PMNs showed a significantly increased transmigration across the human choroid plexus papilloma cells (HIBCPP) after infection with wild-type *N. meningitidis* (Steinmann et al., 2013). In contrast, a significantly decreased monocyte transmigration rate after bacterial infection of HIBCPP could be observed. Interestingly, in co-culture experiments with monocytes and PMNs, transmigration of monocytes was significantly enhanced. Analysis of the paracellular permeability and transcellular epithelial electrical resistance showed an intact barrier function during the leukocyte transmigration. Further analysis of secreted cytokines/chemokines indicated a distinct pattern after stimulation and paracellular of PMNs and monocytes with the help of different imaging techniques. Moreover, the transmembrane glycoprotein SIRPα was deglycosylated in monocytes, but not in PMNs, after bacterial infection (Ichiyama et al., 2009; Steinmann et al., 2013).

Recent studies showed that the vesicles are enriched for JAM-A, PECAM-1, and CD99, all molecules known to be implicated in paracellular migration. In fact, it is likely that many of the observations described above involving JAM-A, PECAM-1, and CD99 were mediated as part of the LBRC (Mamdouh et al., 2008). During the transmigration process, the membrane from the LBRC is targeted to the EJ where it surrounds the leukocyte and establishes interactions between the respective surface molecules (Mamdouh et al., 2003, 2008). The LBRC membrane is thought to assist the passage of the leukocyte through the EJ by removing structural barriers to transmigration, such as VE-cadherin and associated catenin. The importance of the LBRC can be illustrated by the observation *in vitro*, that disruption of LBRC trafficking to the migration site can block the leukocyte transmigration (Mamdouh et al., 2008). Whilst there is conclusive evidence supporting the involvement of the LBRC and the associated molecules in neutrophils transmigration, aspects of their roles in the overall pathway remain to be defined. In addition, numerous other molecules expressed on the endothelial surface have been implicated in neutrophils transmigration, indicating that many facets of the paracellular migration pathway are yet to be understood (Mueller et al., 2013).

In conclusion, in spite of differences, the paracellular and transcellular migration pathways share many similarities in terms of molecular interactions and mediators. However, it remains unknown about the complete mechanisms that drive both pathways (Wittchen, 2009). Advances in imaging and molecular biology have contributed to the recent significant advancements in the field of leukocyte transmigration. The identification of additional steps in the leukocyte adhesion cascade and the intricacy of established responses such as the transmigration step have been acknowledged (Muller, 2014). Whilst the progress has been made, there are still many aspects of the neutrophils–endothelial relationship that need to be further investigated, such as what factors induce transcellular or paracellular migration of leukocytes. From the former study we conclude that the transmigration of leukocytes across the BBB into the CNS is a critical feature of the pathogenesis of BM (Chi et al., 2012). The benefits of understanding the hallmark feature of leukocyte transmigration could be immense and lead us to the identification of novel pathways and targets that could be utilized in the search for treatments to promote host defense or mitigate BM injury.

## NF-κB signaling pathway: the mechanistic link of the triad and drug target

The NF-kB signal transduction pathway, which is the master regulator of the innate immunity, plays important roles in microbial infections, including the pathogenic triad of BM (Chi et al., 2012). This pathway is activated by a variety of stimuli that occur in BM, including various factors from the host (e.g., cytokines) and pathogens (e.g., LPS, adhesins). It is also a transcriptional activator of many genes involved in the pathogenic triad of BM (e.g., IL–1β, TNF–α, IL-6, IL-8). During the past several decades, evidence has accumulated that deregulated activity of this pathway has been linked to the progression of a number of human diseases, including cancer, BM, rheumatoid arthritis, streptococcal cell wall-induced arthritis, experimental colitis, septic shock, Alzheimer's disease, multiple sclerosis, traumatic CNS injury, and cerebral ischemia (Koedel et al., 2000; de Souza et al., 2011; Anthony Jalin et al., 2015; Bennani-Baiti et al., 2015; Yu et al., 2015). Therefore, the NF-kB signaling pathway should be a good drug target that can hasten development of novel therapeutic agents. NF-kB inhibitors, including N-acetyl-leucinyl-leucinyl-norleucinal (ALLN), Caffeic acid phenethyl ester (CAPE) and BAY 11-7085 (BAY), have been found to reduce the CNS inflammation and to protect rat brains from inflammatory injury following transient focal cerebral ischemia (Khan et al., 2007) and pneumacoccal meningitis (Koedel et al., 2000). ALLN, CAPE, and BAY could inhibit IkB proteolysis, NF-kB binding to the DNA, and IkB phosphorylation, respectively. Preceding infection such as BM has been shown to be associated with poor outcomes after stroke (Anthony Jalin et al., 2015). Simvastatin, a powerful lipid-lowering drug, has been found to reduce inflammatory responses in vascular diseases and brain tissue damage through blocking the nuclear translocation of NF-κB, a key signaling event in expressions of various proinflammatory mediators (Anthony Jalin et al., 2015). Our studies on the host-pathogen interplay have shown that α7 nAChR, an essential regulator of inflammation and immunity, is critical for the pathogenesis and therapeutics of BM (Chi et al., 2011). NF-kB is involved in the regulatory mechanisms of α7 nAChR (Heckenberg et al., 2014). Several studies show that stimulation of nAChRs impairs the host defense against bacterial infections caused by *E. coli, L. pneumophila, C. pneumonia*, GAS, methicillin-resistant *S. aureus* (**MRSA**), and *S. pneumoniae* (Yu et al., 2015). Using the *in vitro*/*in vivo* models of the BBB and RNA-seq, our drug repositioning studies have shown that memantine, a FDA-approved drug for treatment of Alzheimer's disease, could very efficiently block pathogenicities induced by meningitic *E. coli* E44 and IHE2015 (multiple antibiotic resistance strain) in a manner dependent on α7 nAChR (Wang et al., 2015; Yu et al., 2015). These findings suggest that memantine is a promising drug for the treatment of BM with potentially quick application in pediatrics since this drug has been used to treat Alzheimer's disease over 30 years showing acceptable safety profiles and good tolerability (Wang et al., 2015).

## Summary

**Figure 5:**
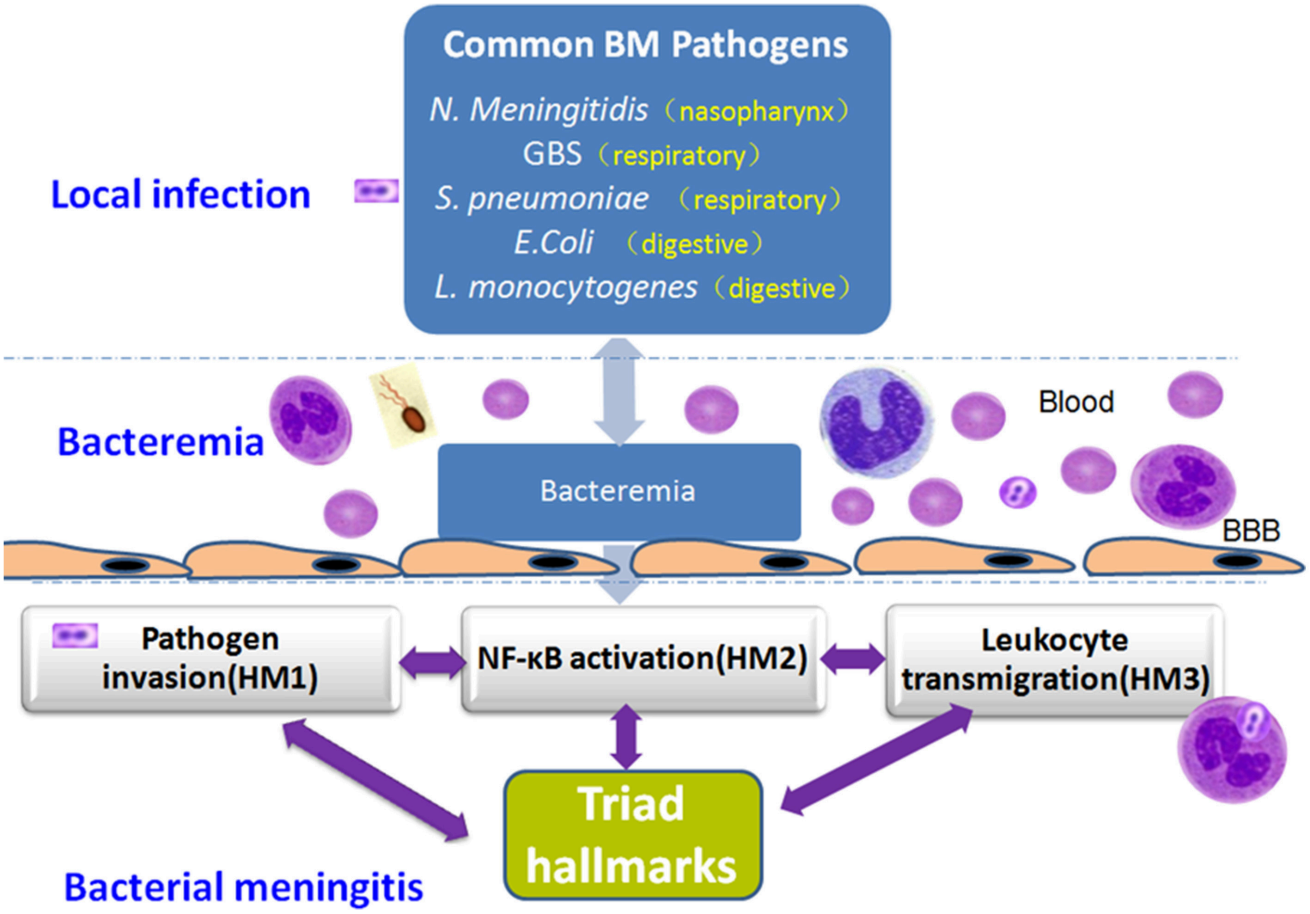
**Mechanistic triad of bacterial meningitis (BM): Pathogen penetration, NF-kB activation and leukocyte transmigration that occur at the BBB**. BM caused by pathogens usually begins with local tissue adhesion and colonization of the potentially meningitic microorganisms. After successful traversal of the local tissue barrier (e.g., the gut barrier for enteric pathogens), a high degree of bacteremia is required for penetration of pathogens across the BBB to cause meningitis. NF-kB activation, a mechanistic link, contributes to both pathogen penetration and leukocyte transmigration across the BBB. The NF-kB signaling pathway can serve as a drug-discovery platform.

The unique triad pathogenic features of BM are driven by interactions between meningitic pathogens and the host defense systems. Basic and translational studies have revealed the molecular and cellular mechanisms underlying these interactions in BM, leading to the discovery of the important host factors and bacterial virulence determinants reflecting pathogen penetration, NF-κB activation and leukocyte transmigration that occur at the BBB, which are the THFs of BM (Table 1 and Figure 5). The development of novel therapeutic interventions against the triad features of BM presents a challenge for modern medicine.

**Table 1:** **Summary of pathogenic triad features of bacterial meningitis**.

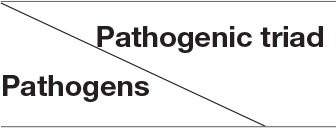	**Bacterial invasion across the BBB**	**PMN.migration across the BBB**	**NF-κB Activation**	**References**
*S. agalactiae* (GBS) *S. pneumoniae*	ACP NanA	Pial venules Choroid plexus EC	LTA and LPT through TLR2. PLY through TLR4	Barreiro et al., 2008; Wittchen, 2009; Hanke and Kielian, 2011; Feng et al., 2014; Landwehr-Kenzel and Henneke, 2014; Simonsen et al., 2014
*E. coli*	GimA	Vim	Vim and PSF	Lee et al., 2005; Che et al., 2011
*N. meningitidis*	Type IV pili NadA	Ezrin and moesin. Neisseria-containing vacuole	Unclear	Abbruscato et al., 2002; Carman and Springer, 2004; Muller, 2011; Coureuil et al., 2012
*L. monocytogenes*	InlA and InlB	Unclear	Nod1 and InI B	Opitz et al., 2006; Gründler et al., 2013; Koopmans et al., 2013
*M. tuberculosis*	VEGF/Hsp65	ILPs structure	NAC and LTA	Hsieh et al., 2010; Dimasi et al., 2013; Zucchi et al., 2013; Francisco et al., 2015

## Ethic statement

The authors state that they have obtained appropriate institutional review board approval or have followed the principles outlined in the Declaration of Helsinki for all animal experimental investigation.

## Author contributions

SW and SH carried out the design and drafting of the manuscript. LZ participated in the drafting of the part pathogen invasion. LP carried out the transmission electron microscopy of meningitic *E. coli* K1. SH, ZG, AJ, and HC conceived of the study, and participated in its design and coordination and helped to draft the manuscript. All authors read and approved the final manuscript.

### Conflict of interest statement

The authors declare that the research was conducted in the absence of any commercial or financial relationships that could be construed as a potential conflict of interest.
